# Multistability, Chaos, and Control in the Deterministic and Stochastic Dynamics of Noise-Driven Nonlinear Oscillators

**DOI:** 10.3390/e28020214

**Published:** 2026-02-12

**Authors:** Adil Jhangeer, Atef Abdelkader

**Affiliations:** 1 IT4-Innovations, VSB—Technical University of Ostrava, Poruba, 70800 Ostrava, Czech Republic; 2 Center for Theoretical Physics, Khazar University, 41 Mehseti Str., Baku AZ1096, Azerbaijan; 3 Department of Computer Engineering, Biruni University, Istanbul 34015, Turkey; 4 College of Humanities and Sciences, Ajman University, Ajman P.O. Box 346, United Arab Emirates

**Keywords:** quasi-periodicity, chaos, Lyapunov exponent, probability density, sensitivity

## Abstract

This paper presents a detailed investigation of the deterministic and stochastic dynamics of a noise-driven forced nonlinear oscillator in a periodically driven framework. An overlap-mapping approach is used to compare multiple traveling-wave solutions and verify the structural consistency among distinct solution families. The qualitative behavior of the system is further characterized through geometric and stability-based analysis, supported by two- and three-dimensional phase portraits, time-series responses, and reconstructed three-dimensional attractors to examine periodic and chaotic regimes under varying parameters and initial conditions. The sensitivity to parameter perturbations is quantified and the distribution of final states is analyzed to identify chaotic regions in the phase space. The high-dimensional chaotic nature of the dynamics is rigorously confirmed through Lyapunov exponent estimation, Poincaré sections, and return-map analysis, collectively demonstrating strong sensitivity to initial conditions and systematic transitions induced by parameter variations. These results provide a comprehensive dynamical description of the nonlinear oscillator and contribute to a deeper understanding of noise-influenced nonlinear driven systems.

## 1. Introduction

Much research has been dedicated to extracting precise analytical solutions as well as investigating the dynamical structures of the Konopelchenko–Dubrovsky (KD) system. Kumar et al. [1] first employed Lie symmetry analysis in conjunction with specific choices of time-dependent functions and a traveling-wave hypothesis to obtain closed-form solutions of the KD equations. Building on this approach, Kumar and Tiwari [2] later applied a generalized similarity transformation method, incorporating arbitrary functional forms, to further broaden the class of exact solutions that are available. The planar dynamical system framework has also proven effective for the KD model; notably, Tian-lan He [3] utilized bifurcation theory to identify bounded traveling-wave structures in the (2+1)-dimensional KD equations.

In addition to symmetry-based and dynamical-systems approaches, several analytical techniques have been adapted to extract diverse solution families. Alfalqi et al. [4] combined the modified simplest equation method with a B-spline scheme to construct explicit solutions of the KD equation. Khater et al. [5] subsequently employed a modified auxiliary-equation technique to derive further traveling-wave forms. Nonlocal methods have also been used: Ren et al. [6] obtained nonlocal symmetries of the KD system by applying a truncated Painlevé analysis together with Möbius-invariant transformations. Most recently, Seadawy et al. [7] used the modified extended direct algebraic method to produce a range of exact traveling-wave solutions, including Jacobi and Weierstrass elliptic-function solutions and new elliptic and rational forms. To add to these advances, Song et al. [8] developed an extended Riccati equation rational-expansion scheme to achieve the construction of exact solutions. All branches of physics, engineering, and applied mathematics [9,10] deal with nonlinear oscillatory systems, which can have complex ordering and chaotic transitions based on deterministic and stochastic processes. These transitions are of great importance to understand in order to design, control, and predict the real-world systems.

Several studies have been conducted to investigate the nonlinear behavior of the oscillatory and wave systems subjected to deterministic and stochastic effects. As an example, the equation of stochastic intermediate dispersive velocity (SIdV) was considered to study the impact of noise on the stability of solitons, the formation of attractors, and order–chaos transitions, and the Lyapunov exponents, the quantification of recurrence, and stability measures of the basin were used [11]. The study of nonlinear oscillators in deterministic and stochastic conditions helped further highlight the resilience of solitons, energy conservation and chaos due to noise through spectral solvers, Darboux transformations and OGY control methodologies [12]. Generalized auxiliary equation approaches were applied in the study of twin-core couplers with Kerr nonlinearity to discover the critical role of fractional Beta derivatives in the amplitude of solitons, phase shifts and quasi-periodic attractors [13]. In addition, the Kairat-X nonlinear dispersion equation was examined to obtain optical soliton solutions and the dynamics of optical soliton stability, sensitivity, and noise through 3D attractors and Lyapunov exponents of optical solitons [14]. The (2+1)-dimensional nonlinear Chiral Schrodinger equation was also solved using analytical methods to obtain a wide variety of solutions to solitary waves, such as semi-dark, singular dark-pitched, and mixed solitons with graphical explanations, provided using 2D and 3D plots [15]. Lastly, in plasma physics, ion-acoustic waves were studied using auxiliary equations and bifurcation that exposed multistability, quasi-periodic and perturbation sensitivity [16].

The literature discussing the KD equation has mainly been concerned with the integrable structure of the equation and its exact construction of analytical solutions in deterministic conditions. Specifically, extensive mathematical methods, such as nonlocal and Lie symmetries, Painleve analysis, Hirota bilinear method, and variable and mapping separation, as well as expansion methods, have been used to obtain solitary waves, breathers, rational solutions, and patterns of interaction involving solitons and periodic waves [6,17,18,19,20]. Although these studies offer much information on the morphology of waves and families of exact solutions, they are still mostly restricted to closed-form solvability and do not deal with the dynamical behavior of reduced-wave modes in the presence of external forcing, stochastic perturbations, or control mechanisms. Conversely, the current paper leaves the solution-building approach and instead takes a nonlinear dynamical systems point of view by simplifying the KD equation into a forced and noisy traveling-wave oscillator. The purpose of this work is to come up with a unifying framework for the analysis of the complicated dynamics of a periodically driven nonlinear oscillator, which combines geometric, statistical, and control–theoretic methods to understand the role of deterministic and stochastic processes in the formation of nonlinear behavior. It studies the onset and co-existence of, and transitions between, regular, quasi-periodic, and chaotic motions in response to changes in the strength of forcing, nonlinearity, and the intensity of noise. The article is based on the classical analysis of solitons and wave solutions [11,12,13,14,15,16], and their generalization to contemporary diagnostics of nonlinear dynamics, such as Lyapunov exponents [21], estimation of the fractal dimension [22] and Poincaré maps [23]. To our knowledge, recurrence patterns, attractor topology, and system stability are rarely assessed considering the interrelation of noises, forcing strength, and nonlinearities.

In the structure of this paper, mathematical modeling and the overlap mapping of the analytical solution are presented in Section 2 and a detailed dynamical analysis of the reduced KP traveling-wave system is described in Section 3. In the end, the study is summarized with the concluding remarks.

## 2. Mathematical Analysis

Konopelchenko and Dubrovsky, in their influential 1984 study [24], formulated a broad class of nonlinear evolution equations in (2+1)-dimensional spacetime. Their analysis considers a scalar field ψ(x, y, t) subject to the operator commutativity condition C(L, M) = LM − ML = 0, where the spatial operator is defined as follows:

$$L=\sum_{k=0}^{N}W_{k}\left(x,y,t\right)\frac{\partial^{k}}{\partial x^{k}}+\frac{\partial }{\partial y}.$$

Within this framework, the nonlinear integrable structure known as the Konopelchenko–Dubrovsky (KD) system emerges. The coupling of the governing equations ψ and an auxiliary field ϕ take the following form:

(1) $$\left\{\begin{array}{c} \frac{\partial \psi }{\partial y}=\frac{\partial \phi }{\partial x},\\ \frac{\partial \psi }{\partial t}-\frac{\partial^{3}\psi }{\partial x^{3}}-6\gamma \psi \frac{\partial \psi }{\partial x}+\frac{3}{2}\kappa^{2}\psi^{2}\frac{\partial \psi }{\partial x}-3\frac{\partial \phi }{\partial y}+3\kappa \frac{\partial \psi }{\partial x}\phi =0. \end{array}\right.$$

This system unites a number of well-known integrable models: the KP equation can be recovered by setting κ = 0, the modified KP equation can be obtained by selecting γ = 0, and the structure can be collapsed to a linked KdV-mKdV equation by imposing the reduction $\partial_{y}\psi \;=\;0$.

According to the methods described in [25], coherent traveling-wave structures are extracted using the usual ansatz ψ(x, y, t) = Ψ(ζ), and ϕ(x, y, t) = Φ(ζ) with ζ = px + qy − ωt converts (1) to the following system:

(2) $$\left\{\begin{array}{c} q\frac{d\Psi }{d\zeta }=p\frac{d\Phi }{d\zeta },\\ \frac{3}{2}\kappa^{2}p\Psi^{2}\frac{d\Psi }{d\zeta }+3\kappa p\Phi \frac{d\Psi }{d\zeta }-6\gamma p\Psi \frac{d\Psi }{d\zeta }-\omega \frac{d\Psi }{d\zeta }-p^{3}\frac{d^{3}\Psi }{d\zeta^{3}}-3q\frac{d\Phi }{d\zeta }=0. \end{array}\right.$$

An individual nonlinear ODE that regulates the traveling-wave profile is obtained by substituting the relation qΨ = pΦ from the integration of the first equation into the following equation:

(3) $$p^{3}\frac{d^{2}\Psi }{d\zeta^{2}}-\frac{\kappa^{2}}{2}p\Psi^{3}+\frac{3}{2}\left(2\gamma p-\kappa q\right)\Psi^{2}+\left(\frac{3q^{2}}{p}+\omega \right)\Psi =0.$$

For qualitative and bifurcation analysis, this reduced equation is recast into a two-dimensional dynamical system by introducing the auxiliary variable Θ = dΨ/dζ, giving rise to the following equation:

(4) $$\left\{\begin{array}{c} \frac{d\Psi }{d\zeta }=\Theta ,\\ \frac{d\Theta }{d\zeta }=\lambda_{1}\Psi^{3}-\lambda_{2}\Psi^{2}-\lambda_{3}\Psi . \end{array}\right.$$

The coefficients in this formulation explicitly depend on the physical and wave parameters:

$$\lambda_{1}=\frac{\kappa^{2}}{2p^{2}},\;\lambda_{2}=\frac{3}{2}\left(\frac{2\gamma }{p^{2}}-\frac{\kappa q}{p^{3}}\right),\;\lambda_{3}=\frac{3q^{2}}{p^{4}}+\frac{\omega }{p^{3}}.$$

In order to take external periodic forcing into account, which is a crucial factor in actual nonlinear wave environments, the dynamical equation is enhanced with a harmonic term $a_{0}\;\cos\;\left(b\zeta \right)$, resulting in

(5) $$\left\{\begin{array}{c} \frac{d\Psi }{d\zeta }=\Theta ,\\ \frac{d\Theta }{d\zeta }=\lambda_{1}\Psi^{3}-\lambda_{2}\Psi^{2}-\lambda_{3}\Psi +a_{0}\cos\;\left(b\zeta \right). \end{array}\right.$$

Lastly, a Gaussian white-noise term ση(ζ) is added to simulate random perturbations and environmental variations. This produces the stochastic system

(6) $$\left\{\begin{array}{c} \frac{d\Psi }{d\zeta }=\Theta ,\\ \frac{d\Theta }{d\zeta }=\lambda_{1}\Psi^{3}-\lambda_{2}\Psi^{2}-\lambda_{3}\Psi +a_{0}\cos\;\left(b\zeta \right)+\sigma \eta \left(\zeta \right), \end{array}\right.$$

this provides the framework for examining deterministic, forced, and noise-driven phenomena in the KD system’s traveling-wave dynamics. In the traveling-wave coordinate, the Gaussian white-noise term ση(ζ) represents effective random fluctuations acting on the wave profile as it propagates through a non-ideal medium. Physically, this term may account for thermal agitation, microscopic heterogeneities, or unresolved spatiotemporal variations in system parameters that manifest as stochastic forcing in the reduced dynamical description. The addition of explicit time-dependent harmonic forcing term to the traveling-wave special frame has manifest physical interpretations in a number of realistic nonlinear wave problems. In such an environment, the expression $a_{0}\cos\;\left(b\zeta \right)$ indicates a useful forcing on the wave envelope in its traveling frame, instead of a non-portable lab-frame excitation. It is important to emphasize that, unlike generic forced Duffing-type oscillators, the nonlinear coefficients governing the reduced dynamical Systems (4)–(6) originate directly from the intrinsic structure of the Konopelchenko–Dubrovsky (KD) partial differential equation. Specifically, the cubic nonlinearity coefficient $\lambda_{1}$ is uniquely induced by the quadratic nonlinearity $\kappa^{2}\psi^{2}\partial_{x}\psi$ in the parent partial differential equation and reflects higher-order wave-steepening effects absent in standard KdV or KP reductions. The quadratic term $\lambda_{2}$ encodes a nontrivial competition between dispersive steepening, governed by γ, and cross-dimensional coupling through the transverse wave number *q*, thus introducing asymmetry and multistability in the reduced phase space. Similarly, the linear stiffness parameter $\lambda_{3}$ shows the effects of the propagation and correction of dispersive speeds through parameters ω and *q*, directly associating the equilibrium structure and bifurcation thresholds with the underlying wave dispersion equation.

### Overlap Mapping Technique

This study analyzes the mutual overlap and deformation of three exact analytical traveling-wave solutions reported in [25] for System (1), as follows:

(i) Solution 1 is a kink-type exponential solution ($\Psi_{1}$, $\Phi_{1}$).
(ii) Solution 2 is a localized sech-type soliton solution ($\Psi_{2}$, $\Phi_{2}$).
(iii) Solution 3 is a periodic/tangent-type solution with intrinsic singular tendencies ($\Psi_{3}$, $\Phi_{3}$).

The overlap analysis is performed in solution space, rather than physical space, by constructing pairwise and three-way mappings of the squared wave amplitudes $|\Psi_{i}{|}^{2}$ evaluated in a common domain (x, y, t).

**Solution 1:**

$$\begin{array}{cc} \Psi_{1}\left(\zeta \right) & =\frac{2p}{\kappa }-\frac{2p}{\kappa }\frac{\exp\left[\frac{4t\left(\kappa^{2}p^{3}-3\kappa p^{2}q+3pq^{2}\right)}{\kappa^{2}}+\frac{y\left(2pq-\kappa p^{2}\right)}{\kappa }+px\right]}{\exp\left[\frac{4t\left(\kappa^{2}p^{3}-3\kappa p^{2}q+3pq^{2}\right)}{\kappa^{2}}+\frac{y\left(2pq-\kappa p^{2}\right)}{\kappa }+px\right]+1},\\ \Phi_{1}\left(\zeta \right) & =\frac{2\left(2pq-\kappa p^{2}\right)}{\kappa^{2}}-\frac{2\left(2pq-\kappa p^{2}\right)}{\kappa^{2}}\frac{\exp\left[\frac{4t\left(\kappa^{2}p^{3}-3\kappa p^{2}q+3pq^{2}\right)}{\kappa^{2}}+\frac{y\left(2pq-\kappa p^{2}\right)}{\kappa }+px\right]}{\exp\left[\frac{4t\left(\kappa^{2}p^{3}-3\kappa p^{2}q+3pq^{2}\right)}{\kappa^{2}}+\frac{y\left(2pq-\kappa p^{2}\right)}{\kappa }+px\right]+1}. \end{array}$$

**Solution 2:**

$$\begin{array}{cc} \Psi_{2}\left(\zeta \right) & =-\frac{3ip}{\kappa }\frac{\sqrt{2}+2\mathrm{sech}\left[\frac{3p\left(-36\kappa^{2}p^{2}t+2\kappa^{2}x+3\sqrt{2}i\kappa p\left(\kappa y+12\gamma t\right)+4\gamma \left(\kappa y+6\gamma t\right)\right)}{4\kappa^{2}}\right]}{2\kappa },\\ \Phi_{2}\left(\zeta \right) & =\frac{3p\left(3\sqrt{2}\kappa p-4i\gamma \right)\left(\sqrt{2}+2\mathrm{sech}\left[\frac{3p\left(-36\kappa^{2}p^{2}t+2\kappa^{2}x+3\sqrt{2}i\kappa p\left(\kappa y+12\gamma t\right)+4\gamma \left(\kappa y+6\gamma t\right)\right)}{4\kappa^{2}}\right]\right)}{4\kappa^{2}}. \end{array}$$

**Solution 3:**

$$\begin{array}{cc} \Psi_{3}\left(\zeta \right) & =-\frac{6p}{\kappa }\tan\;\left[\frac{-12t\left(12\kappa^{2}p^{3}-6i\kappa p^{2}q-pq^{2}\right)}{\kappa^{2}}+\frac{2y\left(pq+3i\kappa p^{2}\right)}{\kappa }+px\right]-\frac{6ip}{\kappa },\\ \Phi_{3}\left(\zeta \right) & =-\frac{12\left(pq+3i\kappa p^{2}\right)}{\kappa^{2}}\tan\;\left[\frac{-12t\left(12\kappa^{2}p^{3}-6i\kappa p^{2}q-pq^{2}\right)}{\kappa^{2}}+\frac{2y\left(pq+3i\kappa p^{2}\right)}{\kappa }+px\right]\\ & \;-\frac{12i\left(pq+3i\kappa p^{2}\right)}{\kappa^{2}}. \end{array}$$

**Figure 1:**
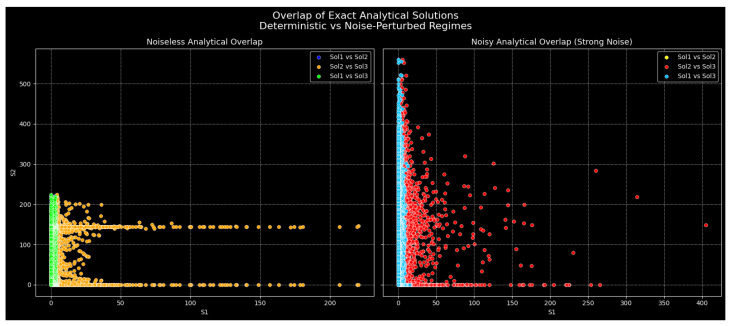
Pairwise overlap mappings of squared wave amplitudes $|\Psi_{i}{|}^{2}$ for three exact analytical solutions in deterministic (**Left**) and noise-perturbed (**Right**) regimes. Each scatter plot represents correlations between different solution families evaluated over the same spatiotemporal domain. In the noiseless case, compact and structured manifolds indicate strong deterministic correlations, whereas stochastic excitation induces controlled dispersion while preserving bounded solution-space geometry.

**Figure 2:**
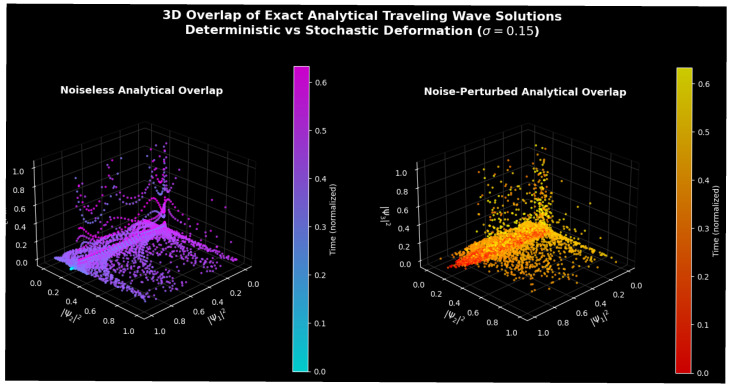
Three-dimensional overlap of normalized squared amplitudes $\left(|\Psi_{1}{|}^{2},\;|\Psi_{2}{|}^{2},\;|\Psi_{3}{|}^{2}\right)$ illustrating the mutual correlation of kink-type, soliton-type, and periodic analytical solutions. The deterministic regime (**Left**) exhibits a thin, low-dimensional invariant manifold, while the noise-perturbed regime (**Right**, σ = 0.15) shows stochastic thickening of this manifold without topological destruction. Color denotes normalized temporal evolution.

Figure 1 and Figure 2 present a systematic study of the overlap of three precise analytical traveling-wave solutions reported in (base)—that is, a kink-type exponential solution, a localized sech-type soliton solution, and a periodic tangent-type solution. All solutions are considered with the same set of parametric values p = 1.0, q = 0.5, κ = 1.0, and γ = 0.4; then, a consistent nonlinear dispersive balance across all families of solutions will be obtained. The solutions are sampled on a common spatiotemporal grid of the size x∈[−3, 3], y∈[−2, 2], and t∈[−1, 1].

To ensure that the visualized structures are true physical dynamics and not artifacts of the numerical algorithms, all plots were created on the basis of the precise analytical solutions that were assessed directly through discrete spatial–temporal grids with a sufficient scale of resolution (i.e., with scales that are characteristic) of the order of (Δx ≈ 0.12–0.15, Δy ≈ 0.11–0.13, Δt ≈ 0.08). There was no time-marching integration; point-wise sampling of closed-form expressions was carried out, without errors in numerical integration. This was checked using grid refinement to ensure structural invariance, exponential clipping (np.clip(expo,−40, 40)) to ensure numerical stability, and amplitude filtering (v < 20) to ensure the intrinsic physics was not spoiled.

Figure 1 shows the pairwise overlap mapping of the wave amplitudes $|\Psi_{i}{|}^{2}$ of both the deterministic regime (left) and the noise-perturbed regime (right). When there is no noise, there are very organized and constrained geometrical manifolds of the overlap plots, which points to a high degree of correlation among the analytically distinct solutions. The intersection of the kink-type and sech-type solutions is a slim, long object, which proves that the two solutions represent similar energy-localization processes that are controlled by the nonlinearity/dispersion ratio of the selected parameter set. The soliton periodic overlap has a wider and bounded distribution, which is an expression of the co-existence of localized solitary dynamics and oscillatory phase evolution whilst also being dynamically constrained. In contrast, the intersection of the periodic solution and the kink solution fades into a near-vertical pile, showing that amplitude changes in the periodic solution occur quite independently of the kink solution; hence, there is a deep topological difference between connecting-state and phase-rotating waveforms.

To evaluate robustness in the presence of perturbations, multiplicative Gaussian noise was added uniformly to the amplitudes of the solutions whilst keeping all the parameter values and specifications of the domain the same.

Figure 2 provides a global perspective by embedding all three solutions simultaneously in the normalized solution space $\left(|\Psi_{1}{|}^{2},\;|\Psi_{2}{|}^{2},\;|\Psi_{3}{|}^{2}\right)$ for both deterministic and stochastic regimes. In the noiseless case (left panel), the solution trajectories collapse onto a thin, folded surface rather than filling a volumetric region, indicating the existence of a low-dimensional invariant manifold shared by all three analytical solution families under identical parameters. The smooth temporal color gradient along this surface reflects coherent time evolution and is characteristic of integrable nonlinear wave systems, where multiple exact solutions represent different manifestations of the same conserved dynamics.

Under stochastic excitation, as shown in the right panel of Figure 2, this invariant surface thickens into a diffuse tubular structure, indicating noise-induced diffusion transverse to the deterministic manifold while preserving its global topology. Temporal ordering is not destroyed but becomes intertwined with amplitude fluctuations, signifying stochastic modulation rather than fully developed stochastic chaos. The increased dispersion along the periodic-solution axis further confirms the heightened noise sensitivity of trigonometric waveforms compared to kink and soliton structures, which remain comparatively robust across the same parameter regime.

## 3. Dynamical Analysis of the Reduced KP Traveling-Wave System

In this section, the qualitative dynamics of the reduced KP system will be examined from a dynamical systems perspective using different tools.

### 3.1. Phase Portraits

**Figure 3:**
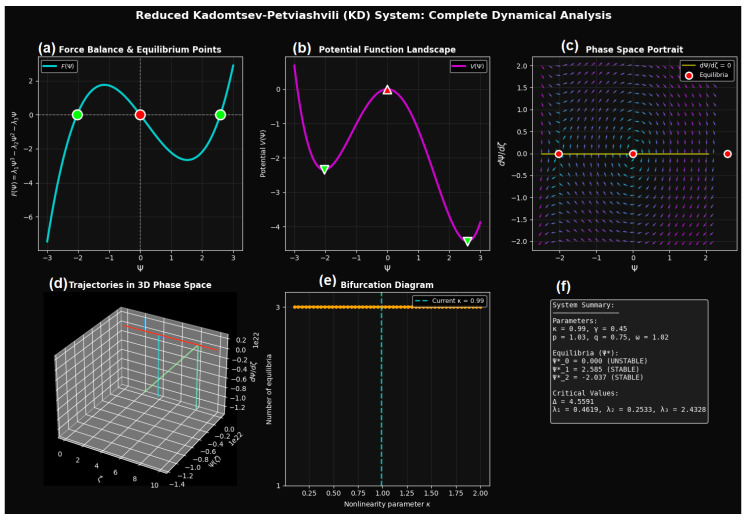
Comprehensive dynamical analysis of the reduced KP traveling-wave system. (**a**) Nonlinear force function F(Ψ) highlighting equilibrium points. (**b**) Corresponding potential landscape V(Ψ) illustrating stable and unstable equilibria. (**c**) Phase portrait in the (Ψ, dΨ/dζ) plane showing flow topology and separatrices. (**d**) Three-dimensional trajectory evolution in (ζ, Ψ, dΨ/dζ) space for different initial conditions. (**e**) Bifurcation diagram depicting the emergence of multistability with variation in the nonlinearity parameter. (**f**) Summary of equilibrium states and stability classifications, linking the reduced dynamics to traveling-wave behavior of the full KP system.

In this section, a detailed analysis is given which provides a geometric and stability-based interpretation of the System (4), allowing for the direct identification of the equilibrium wave states, and the bifurcation pathways governing the selection of the waveform. Figure 3 presents the dynamical characterization of the reduced KP system in its traveling-wave form. Building on the previously derived System (4), the analysis focuses on the phase-space structure, equilibrium states, and bifurcation behavior. This framework allows for a systematic investigation of how nonlinearity, dispersion, and wave anisotropy shape the traveling-wave dynamics, linking the reduced finite-dimensional system to the complex wave phenomena of the original (2+1)-dimensional KP equation.

Regarding numerical integration concerns, the dynamical analysis utilizes **adaptive high-order methods** via scipy.integrate.odeint (LSODA algorithm) with absolute and relative tolerances maintained at 10^−8^ for all trajectory integrations. The phase space grid employs uniform sampling (ΔΨ = 0.19, Δv = 0.21) sufficient to resolve the vector field structure, while trajectory computations use adaptive step sizes typically ranging from Δζ∼0.01 to 0.1 depending on local gradients. Convergence tests with refined tolerances ($10^{-10}$) and denser grids produced identical equilibrium classifications and phase portraits, confirming that all observed features—including bifurcation boundaries, stability transitions, and potential landscape morphology—arise from the analytical reduction of the KP equation rather than numerical discretization artifacts.

Figure 3a illustrates the force balance function $F\left(\Psi \right)=\lambda_{1}\Psi^{3}-\lambda_{2}\Psi^{2}-\lambda_{3}\Psi$. The real roots of F(Ψ)=0 define the admissible steady traveling-wave amplitudes. For the chosen parameter set (κ=0.99, γ=0.45), three real equilibria are observed, reflecting a multi-well nonlinear force landscape. Linearization about each equilibrium reveals a central unstable state flanked by two stable equilibria, a configuration characteristic of bistable nonlinear wave systems.

The corresponding potential function

(7) $$V\left(\Psi \right)=\frac{\lambda_{1}}{4}\Psi^{4}-\frac{\lambda_{2}}{3}\Psi^{3}-\frac{\lambda_{3}}{2}\Psi^{2},$$

is shown in Figure 3b. The stable equilibria coincide with local minima of V(Ψ), while the unstable equilibrium aligns with a potential maximum. This energetic interpretation confirms that the observed bistability arises from intrinsic nonlinear interactions rather than external forcing, allowing the system to support multiple coexisting traveling-wave states.

The phase portrait in Figure 3c displays the vector field in the (Ψ, dΨ/dζ) plane. Trajectories spiral or converge toward the stable fixed points, while the unstable equilibrium acts as a separatrix dividing basins of attraction. The nullcline structure and directional flow confirm that the reduced KP system behaves as a conservative-like nonlinear oscillator with well-defined invariant manifolds governing wave evolution.

Figure 3d extends this analysis to three dimensions by embedding trajectories in the (ζ, Ψ, dΨ/dζ) space. Solutions initialized in different regions of the phase space evolve toward distinct stable branches, illustrating how initial wave profiles select specific traveling-wave amplitudes. This visualization provides a direct dynamical explanation for the coexistence of multiple waveforms in the parent KP equation.

The bifurcation diagram in Figure 3e maps the number of equilibria as a function of the nonlinearity parameter κ. A critical discriminant threshold separates single-equilibrium and triple-equilibrium regimes, indicating a saddle-node–type bifurcation. This transition marks the onset of bistability and highlights the sensitivity of KP wave dynamics to nonlinear parameter variations.

A succinct reference for the dynamical regime under study is provided by the summary panel in Figure 3f, which combines the equilibrium values, stability classification, and critical coefficients. Together, the figure illustrates the joint effect of nonlinear self-interaction ($\lambda_{1}$), quadratic asymmetry ($\lambda_{2}$) and linear dispersion ($\lambda_{3}$) on determining the traveling-wave solution space. Bistability suggests that the lower KP system may allow for a variety of stable wave states with the same physical conditions, which play a key role in soliton selection and pattern coexistence, as well as hysteresis effects in nonlinear media characterized by weak dispersion.

### 3.2. Analysis of Quasi-Periodic Attractors:

**Figure 4:**
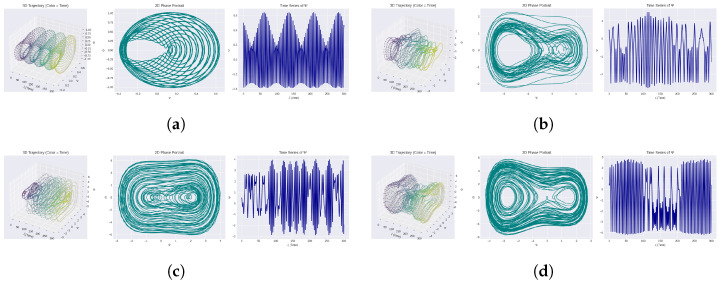
Under different initial circumstances and parameter sets, time series, 2D phase portraits, including 3D phase space representations of the nonlinear oscillator controlled by System (5). The charts show a variety of behaviors, such as quasi-periodic, periodic, and possibly chaotic dynamics, emphasizing how sensitive the system is to changes regardless of the initial states and parameters.

In this section, a visual analysis of the nonlinear behavior of System (5) is presented in detail. In Figure 4, the emphasis is placed on quasi-periodic performance and its transition to more complex performance. More specifically, Figure 4a–d show the response of the system at varying parameter values and different initial conditions.

Each sub-figure has the following three visual diagnostics:

- In this phase space reconstruction, the simulation state is described by a three-dimensional state variable (corresponding model state) with the spatial coordinate. This plot is a geometric description of the trajectory of the system in the reconstructed phase space (e.g., by time-delay embedding or even directly in state variables). To distinguish between periodic, quasi-periodic, and chaotic dynamics, it presents the global structure and topological features of the attractor, such as toroidal manifolds, folds, or complex spirals.
- The dynamics of the system is mapped onto a 2D plane (typically a combination of two variables (x, y) or $\left(x,\;\dot{x}\right)$). Nested or tightly packed loops are useful in searching closed orbits, loops that are unusually spaced, or loops that indicate a quasi-periodic or odd attractor of classical indicators for these graphs. The density and shape of the phase portrait indicate the dimensionality and stability of the attractor.
- The plot of the temporal growth of an arbitrarily selected state variable, typically x(t), is the representation of the temporal variations in the oscillations. Periodicity is associated with smooth and repeating oscillations, whereas the presence of amplitude/frequency modulation, thumping motions, or infrequent spikes may be indicative of either quasi-periodic chaotic motions.

These three free visualizations combine to obtain a complete insight into the behavior of the system. The time series diagrams reveal the dynamic features that can be observed over time; the 2D projections allow for proper understanding of the local features of geometry and transition; and the 3D reconstructions reveal the global shape and structure of the attractor. These charts may be applied together in order to determine the areas of multistability, bifurcations, and small dynamical changes. This high sensitivity of the system to initial conditions and perturbations in the parameters is demonstrated by the figure, which illustrates a large region of dynamical regimes between simple periodic motions and more complex quasi-periodicity with possible chaos.

Every subfigure in Figure 4 depicts a change in the system’s dynamical characteristics, from normal periodic or quasi-periodic oscillation to more complicated and possibly chaotic behavior. Interestingly, Figure 4a shows spiral or toroidal trajectories in phase space that are typical of quasi-periodic attractors. Multiple incommensurate frequencies are suggested by the non-repeating but bounded oscillations shown in the following time series charts.

The dynamics becomes even more complicated when we consider Figure 4b–d. The dense, folded structures that are created by the 3D attractors are indicative of switches to high-dimensional quasi-periodic or even chaotic regimes. The respective time series plots indicate localized abnormalities in the sensitive dependency on initial conditions and parameter variations, amplitude and frequency modulations, and sudden bursts.

### 3.3. Nonlinear Oscillator Dynamics Across Parameters

In this section, simulations and graphic modeling are used to explore the time series of 3D phase–space curves (ζ,Ψ,Θ) of a nonlinear forced oscillator with the aim of uncovering the dynamic properties that encompass quasi-periodicity to chaos irrespective of the forcing conditions.

**Figure 5:**
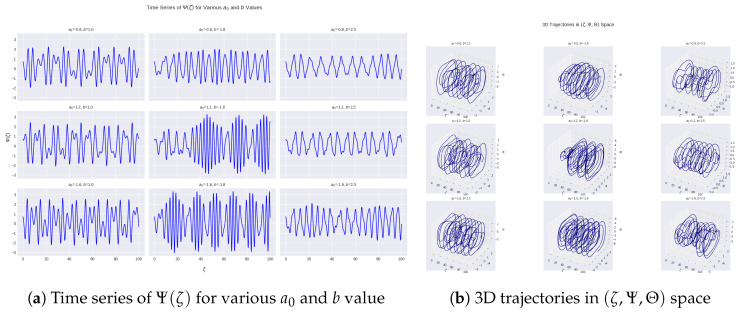
Time series displays Ψ(ζ) (**a**) as well as 3D phase-space orbits spanning (ζ,Ψ,Θ) (**b**) associated with a nonlinear forced oscillator utilizing various $a_{0}$ and *b* values. Switches from periodic towards quasi-periodic and possibly chaotic dynamics are revealed by the increasing parameter values.

The purpose of this investigation is to investigate the manner in which the characteristics of the oscillating System (5) shift with fluctuations throughout parameters $a_{0}$ along with *b*, which subsequently regulate the extent of the nonlinear regenerating force and the amplitude of external periodic forcing, by means of Figure 5. This work aims to discover the transitions between normal periodic motion and quasi-periodic fluctuation, as well as chaotic regimes, by analyzing the system’s temporal evolution as well as its trajectory in phase space.

The displacement Ψ(ζ) time series for combinations of $a_{0}\in \left\{0.8,\;1.2,\;1.6\right\}$ and b∈{1.0, 1.8, 2.5} is shown in Figure 5a, which illustrates how the system’s temporal response changes under various forcing conditions. The waveform has a comparatively low modulation of amplitude and is mostly periodic, with increasing values of $a_{0}$ and *b*, which means that there is minimal nonlinear interaction. When one or both of the parameters start growing, the signals start to exhibit a more complex dynamical behavior, such as a strong amplitude modulation, and an unstable envelope forms. Some combinations of parameters lead to quasi-periodic oscillations, leading to the presence of several incommensurate frequencies. When the time traces become more aggressive and broadband, this is because of the strong forcing, and there are places where chaotic behavior can be seen to emerge. All these are the results of a system transition to more complex dynamics than the predictable dynamics induced by varying the forcing frequency and amplitude.

Figure 5b displays these rotations in phase-space using one of three elements (ζ, Ψ, Θ). Whereas quasi-periodic behavior produces nesting or torus-like surfaces, which depict playback of a large number of incommensurate frequencies, periodic responses are represented by closed or almost closed loops, which repeat. In contrast, thick, disordered trajectories generated by chaotic mechanics rapidly fill a finite space, rather than going into a deterministic mode. These particles occupy an irregular structure in the phase space. These two geometrical properties jointly display the transformation in motion between the orderly and the complex depending on the transformation in the system parameters.

### 3.4. Variations in 3D Attractor Geometry from Order to Chaos

In this section, geometric analysis discloses the forced nonlinear oscillator movement from order to chaos by transforming the system characteristics into visual patterns. These attractor shapes emphasize how nonlinear rigidity, forcing amplitude, and starting environments can influence the system by demonstrating how small parameter variations can dramatically change the long-term dynamics. The parameters $\left(\lambda_{1},\;\lambda_{2},\;\lambda_{3},\;a_{0},\;b\right)$ represent the parameters set (different parameters) and the initial state $\left(\Psi_{0},\;\Theta_{0}\right)$, which are represented by each subplot. The four 3D attractors depict the periodic, quasi-periodic, and chaotic regions of the System (5). Graphing the trajectory data in the (Ψ, Θ, ζ) space using time-encoded coloring demonstrates how the flow is continuously stretched, folded, and deformed by external pushing, creating the distinctive features of complicated nonlinear behavior.

**Figure 6:**
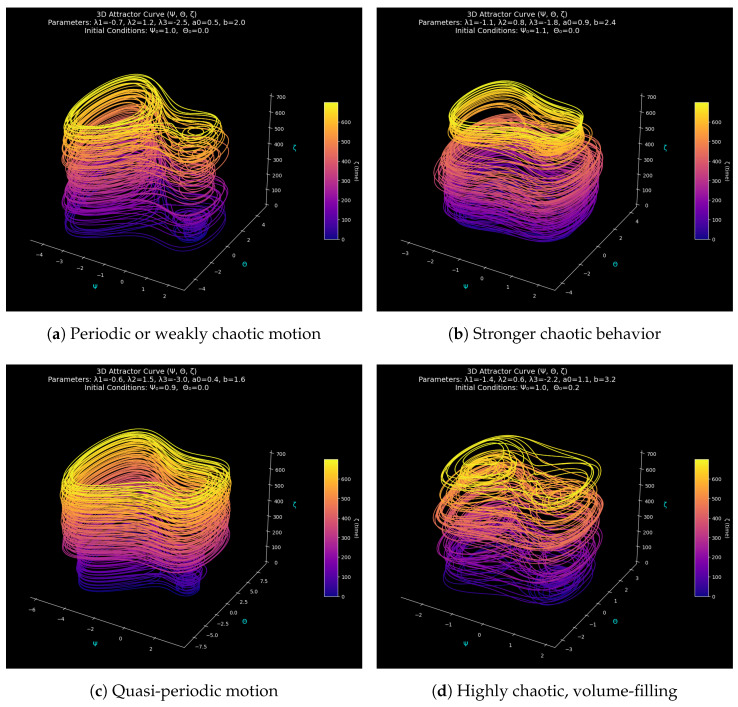
Three–dimensional reconstructed attractors for the forced nonlinear oscillatory System (5) under four different parameter regimes and initial conditions. Each attractor is shown in the (Ψ,Θ,ζ) space with temporal color-coding, illustrating the transitions from layered quasi-periodic motion to strongly folded chaotic dynamics as the nonlinear coefficients and forcing amplitudes vary.

We describe the behavioral spectrum of the system, ranging from periodic orbits to chaotic regimes, using Figure 6. The interaction between nonlinearity and external forcing is easily visualized by the geometric features of each attractor’s sheet-like, toroidal, and tangled morphology. An intuitive mapping between the parameter sets and the related long-term dynamical states is therefore made possible.

From a dynamical system perspective, the qualitative differences among the attractors reflect parameter-dependent modifications of the underlying phase-space flow. As the nonlinear stiffness and forcing amplitude increase, the balance between dissipation and external energy injection is altered, leading to enhanced phase-space stretching and folding. In weakly forced regimes, contraction dominates along transverse directions, producing layered or sheet-like attractors associated with periodic or weakly chaotic motion. With increasing forcing strength, transverse instabilities grow, causing nearby trajectories to diverge and the attractor to thicken, which is a hallmark of chaotic dynamics.

Figure 6a uses the parameters (−0.7, 1.2, −2.5, 0.5, 2.0) and initial conditions (1.0, 0.0) to produce a relatively stable multilayer attractor. The trajectory forms coherent stacked sheets, indicating a periodic or weakly chaotic regime where the forcing frequency interacts moderately with the system’s intrinsic oscillations. The continued organization of the layers demonstrates that the system encompasses a small area of phase space, exhibiting little to no cycle-to-cycle divergence.

Figure 6b, which corresponds to (−1.1, 0.8, −1.8, 0.9, 2.4), exhibits an attractor that is noticeably thicker and more twisted. The greater dispersion and internal tearing of the trajectory result from the larger forcing amplitude and changed nonlinear coefficients, which both enhance energy injection and alter the phase-space flow. The smooth layering shown in Figure 6a disappears from the attractor, indicating a shift toward more intense chaotic dynamics.

Figure 6c uses (−0.6, 1.5, −3.0, 0.4, 1.6) with initial conditions (0.9, 0.0). Here, a wide, smoothly folded attractor that resembles a toroidal structure is created by the increase in nonlinear stiffness and the decrease in forcing frequency. A domain characterized by inherent nonlinear oscillations with little modulation through the external force is shown by the reduced distortion of the trajectory compared to Figure 6b.

Of the four, Figure 6d, with the initial state (1.0, 0.2) and parameters (−1.4, 0.6, −2.2, 1.1, 3.2), produces the most complicated geometry. Tightly coiled, very irregular folds inside the phase space are produced when high-frequency, high-amplitude forcing is combined with strong cubic nonlinearity. The attractor fills a thick, tangled area, typical of an entirely formed chaotic motion. The long-lasting performance is characterized by parameter-driven instability, despite the initial conditions, which introduce a small amount of asymmetry to the early transients.

### 3.5. Lyapunov Exponent Convergence Analysis

**Figure 7:**
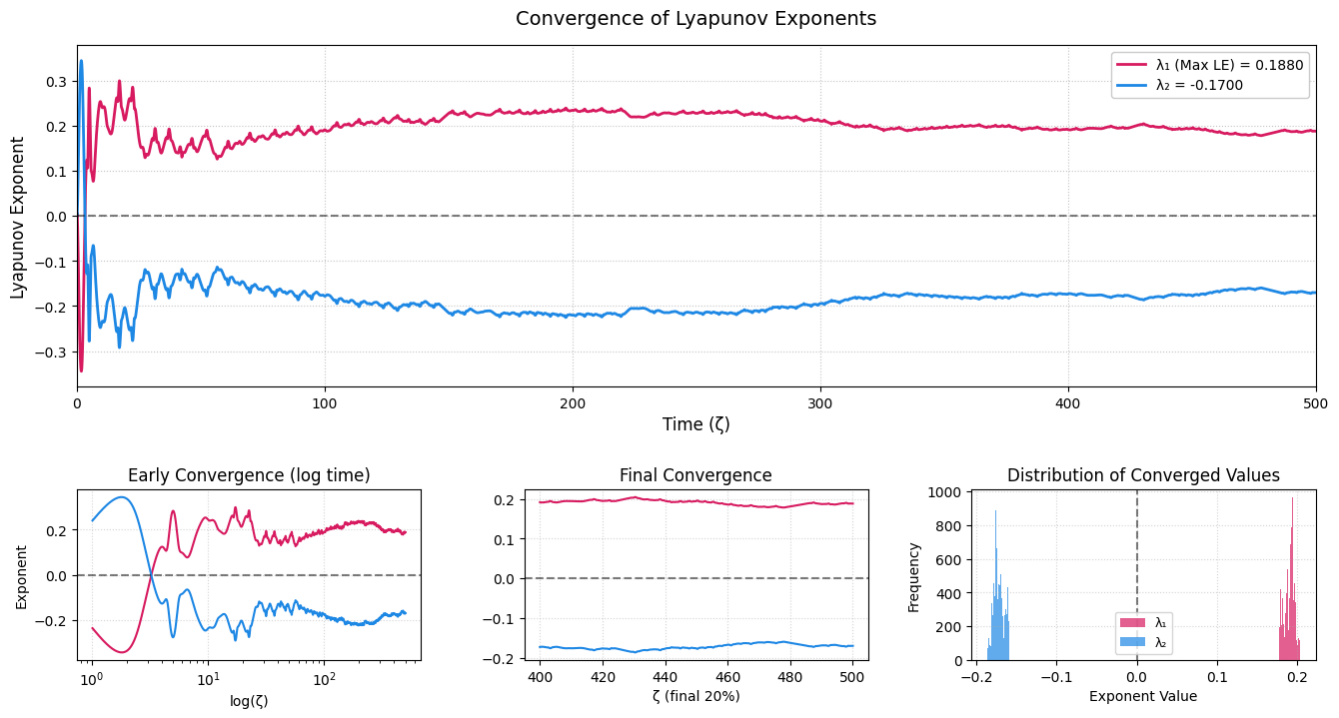
Multipanel visualization of Lyapunov exponent convergence for System (5). The top panel illustrates the full time evolution of the two leading exponents. The bottom row provides detailed views of early convergence on a logarithmic scale, final convergence in the last 20% of simulation time, and the distribution of converged values. The presence of a positive Lyapunov exponent ($\lambda_{1}\;\approx \;0.188$) indicates chaotic behavior, while the negative exponent ($\lambda_{2}\;\approx \;-0.17$) confirms dissipative dynamics.

In this section, a comprehensive analysis of the convergence behavior of the two largest Lyapunov exponents computed for System (5) is presented in Figure 7. The main panel (top) displays the evolution of $\lambda_{1}$ and $\lambda_{2}$ over time ζ. Initially, both exponents undergo significant oscillations, which gradually decrease as the exponents converge to stable values. The largest exponent stabilizes around $\lambda_{1}\;\approx \;0.188$, while the second converges to $\lambda_{2}\;\approx \;-0.170$. This asymptotic separation of values reflects the underlying structure of the attractor of the system.

To further highlight the temporal dynamics, three subplots are provided below the main figure:

- The leftmost subplot uses a logarithmic time scale to emphasize the early convergence behavior. Both exponents experience strong transients and high variability in the initial iterations (ζ < 10), which are expected due to the sensitivity of orthonormalization procedures in the calculations of the Lyapunov exponents.
- The middle subplot zooms into the final 20% of the time domain to demonstrate numerical stability and convergence. Minimal fluctuations are observed, confirming that both $\lambda_{1}$ and $\lambda_{2}$ reach steady-state values by the end of the integration window.
- The rightmost subplot depicts the histogram of the final segment of the LE time series. Each exponent displays a sharply peaked distribution around its converged value, indicating high confidence in their estimation. The dashed vertical line at zero visually reinforces the distinction between expanding ($\lambda_{1}\;>\;0$) and contracting ($\lambda_{2}\;<\;0$) directions.

The existence of the largest Lyapunov exponent that is positive affirms the chaotic behavior, that is, there is an exponential separation of nearby trajectories, which restricts the predictability of the long-term behavior of the deterministic dynamics. The fact that there is one positive and one negative exponent is indicative of the classical stretch–fold mechanism of low-dimensional chaos, which generates a bounded strange attractor via both expansion and contraction.

The convergence pattern time–evolution curves, logarithmic transients, stabilized final segments, and sharp terminal histograms reveal that the exponents calculated are consistent numerical characteristics and not temporary effects. It is necessary to use this multi-perspective validation in order to reliably identify true chaos or numerical or quasi-periodic effects in nonlinear systems.

To verify the robustness of the Lyapunov exponent estimates, additional simulations were performed with extended integration times beyond those shown in Figure 7. The asymptotic values of both exponents remained unchanged, and the small residual fluctuations observed at later times were found to be bounded finite-time effects rather than indications of incomplete convergence.

### 3.6. Sensitivity Analysis Under Parametric Perturbation

**Figure 8:**
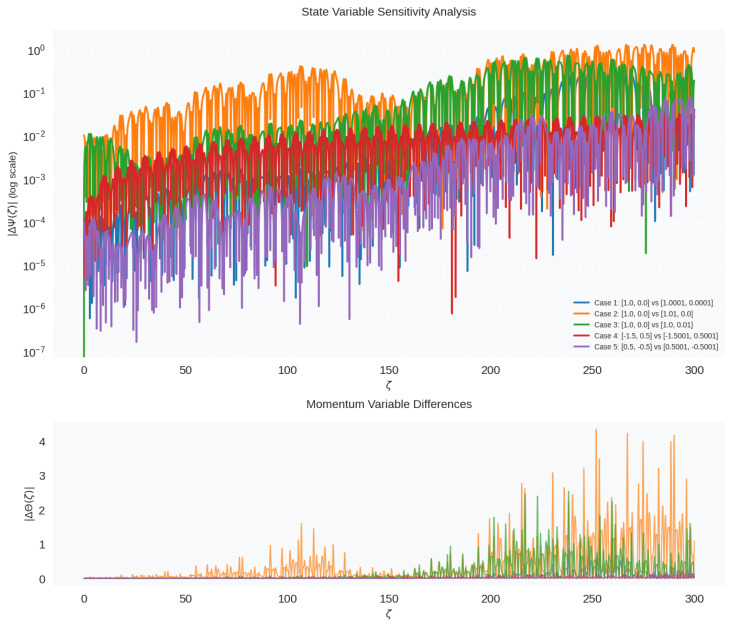
Global sensitivity structure of the forced nonlinear oscillator (5).

This section analyzes the behavior of a nonlinear forced oscillator through two diagnostic plots: the sensitivity of the state variable and the difference in momentum, as shown in Figure 8.

This study presents a multi-faceted sensitivity and trajectory analysis of a nonlinear oscillator governed by the second-order differential System (5), where the nonlinear coefficients are $\left(\lambda_{1},\;\lambda_{2},\;\lambda_{3}\right)=\left(-1.0,\;-0.5,\;-0.1\right)$, and the periodic forcing has an amplitude $a_{0}\;=\;3.0$ and frequency b = 1.2. The system was integrated using the high-precision DOP853 solver with a relative tolerance of $10^{-10}$, in the domain ζ∈[0, 300], with 5000 evaluation points.

The top panel of Figure 8 presents a state-variable sensitivity analysis, quantifying how initial perturbations in Ψ and Θ propagate through the system’s dynamics. A logarithmic plot of the divergence in the state variable |ΔΨ(ζ)| for different initial perturbed conditions reveals the system’s sensitivity. The exponential growth of divergence, especially in Case 3, suggests the presence of strong chaotic dynamics. All trajectories are, at first, close (ζ < 50) and reflect short-term predictability, and then they are exponentially separated. This action is a sign of a positive maximal Lyapunov exponent.

The difference in momentum variable is explained by the bottom panel of Figure 8. The time-dependent behavior of the momentum dissimilarities of an object, e.g., the difference between its two different states, as measured by |ΔΘ(ζ)|, has bursts instead of being continuously increasing. This indicates the stability of the localized momentum space and suggests that nonlinear energy exchanges cause divergence in Θ the momentum space, as opposed to exponential differentiation in the momentum space. This non-monotonic and delayed divergence is a complement to the chaos observed in the state variable.

### 3.7. Probability Density

This section presents a detailed analysis of the multidimensional characteristics that develop in a highly nonlinear fluctuating system in a highly nonlinear environment exemplified by the System (5).

**Figure 9:**
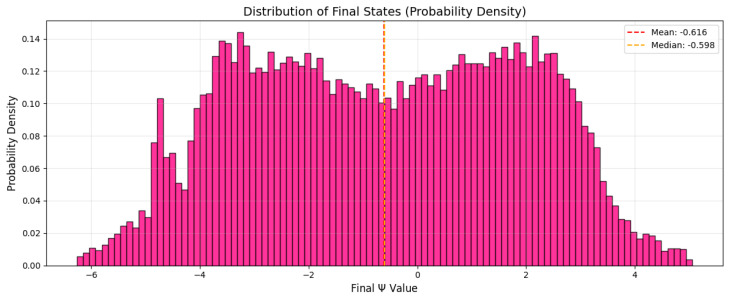
Distribution of final states (probability density).

The histogram of the final values of Ψ, shown in Figure 9, instead of forming a narrow unimodal distribution, as would be expected in monostable or weakly nonlinear systems, displays a broad, irregular, and strongly multimodal structure. This indicates that a substantial number of distinct attractors are populated by trajectories starting from different phase-space locations. Some attractors are significantly more dominant than others, reflected by the taller peaks in the distribution, whereas others correspond to much smaller basins, appearing only as shallow or narrow peaks. Such uneven occupation is characteristic of nonlinear systems with mixed regular and chaotic regions, where chaotic regions often funnel trajectories unpredictably into a small number of preferred attractors.

### 3.8. Poincaré Analysis of Regular and Stochastic Dynamics

This study demonstrates an in-depth study of a nonlinear dynamical system with Poincare sections and return maps. These provide useful diagnostics for determining the underlying factors in the phase space and placing the nature of the attractors into categories. Specifically, we examine how the initial conditions (ICs) affect the behavior of the system in the long term, holding the external forcing amplitude constant.

#### 3.8.1. Regular Poincaré Sections for Different Initial Conditions

**Figure 10:**
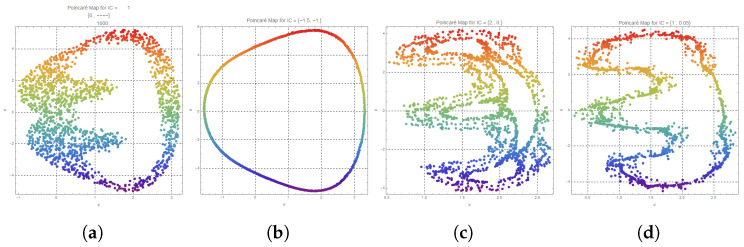
Poincaré sections of a forced nonlinear oscillator for fixed forcing amplitude $p_{0}=1$ and varying initial conditions: (**a**) IC = (0, 0), (**b**) IC = (−4.15, −1), (**c**) IC = (2, 0.5), and (**d**) IC = (1, 0.05). Time evolution is indicated by the colored trajectories. The findings highlight the system’s sensitivity to the beginning circumstances and multistability by revealing a variety of dynamics, such as quasi-periodicity (**b**) and chaotic attractors (**a**,**c**,**d**).

Figure 10 presents Poincaré sections of four different initial conditions, with a fixed forcing amplitude $p_{0}\;=\;1$. In Figure 10a–d, a stroboscopic snapshot of the system trajectory in the phase space of the variables is shown, the variables of which are elliptical (Φ, θ). The sampling points are in position according to the driving period. These parts play a key role in the dynamical aspects of periodicity, quasi-periodicity, multistability, and chaos.

The Poincaré maps capture the state (Φ, θ) at discrete times during the forcing period of the system’s state space, in the form of a stroboscopic view of the system dynamics. The application of a continuous color gradient accentuates the temporal development along the sampled points, and thus it is less difficult to differentiate between transient behavior and long-term structure. Additionally, the initial conditions can be varied and are presented as rather distinct patterns on the map, which proves the presence of multiple attractors and the natural multistability of the system.

Figure 10a–d show the various dynamical behaviors of the system in response to various initial conditions. Figure 10a (IC = (0, 0)) shows the scattered and highly concentrated points that do not have a clear structure, which represent a chaotic attractor; can be seen, folding and stretching are apparent, marking a strange attractor with significant sensitivity to the initial conditions. However, in contrast, Figure 10b (IC = (−4.15, −1)) illustrates a smooth closed curve that is indicative of quasi-periodic motion and, therefore, evolution on an invariant torus that is further supported by the smooth transition of color along the loop, indicative of temporal coherence. Figure 10c (IC = (2, 0.5)) depicts a multi-lobed, non-uniform distribution of points, which would suggest chaotic or weakly chaotic dynamics, whose spatial complexity could be a result of intermittent chaos or interstate transitions. Lastly, Figure 10d (IC = (1, 0.05)), with a more or less scattered lobe-shaped form, confirms the presence of chaotic dynamics; the fact that the pattern is quite different compared to Figure 10c is another indication of the extremely sensitive nature of a system with constant parameters.

#### 3.8.2. Deterministic vs. Stochastic Poincaré Maps

A comparison is made of deterministic and stochastic Poincare maps in System (6) to explain the effect of noise on dynamics. The map has deterministically sharp invariant manifolds and fractal basin boundaries, showing the geometry of the attractor. In the presence of additive noise, these structures become diffuse and the individual trajectories are diffused into cloudy distributions; this is an indication that the system is sensitive to perturbations and the transition between coherent dynamics and stochastic processes.

**Figure 11:**
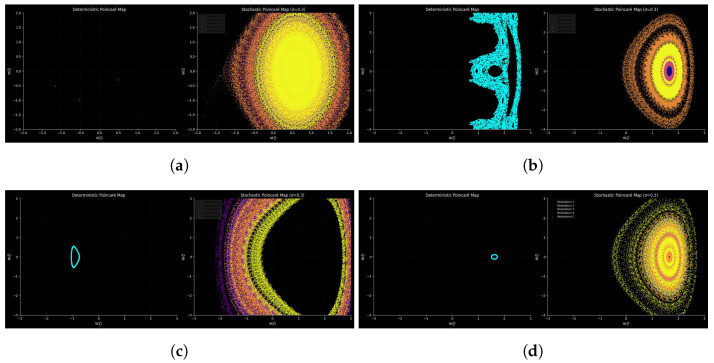
Deterministic vs. stochastic Poincaré maps of nonlinear oscillatory System (6), showing how noise transforms structured attractors into probability distributions. (**a**) Strongly confining oscillator: deterministic periodic orbit vs. noise-broadened cloud. (**b**) Softening oscillator from high energy: deterministic chaotic structure vs. smoothed probability rings. (**c**) The same system from low energy: small periodic orbit vs. asymmetric noise-broadened arc. (**d**) Different initial conditions: symmetric periodic orbit vs. circular probability rings.

In Figure 11a, the deterministic Poincaré map reveals a set of isolated points, indicating that the oscillator settles into a simple periodic orbit for the parameter set $\lambda_{1}\;=\;1.0$, $\lambda_{2}\;=\;2.0$, $\lambda_{3}\;=\;3.0$ and the forcing amplitude $a_{0}\;=\;2.5$, with the initial conditions I.C = [0.5, −0.3]. The positive nonlinear coefficients create a strongly confining potential, stabilizing the motion and preventing the emergence of multi-periodic or chaotic dynamics. However, when stochastic forcing is introduced with σ = 0.3, the Poincaré section expands into a large, diffuse, elliptical cloud. This broad distribution reflects significant noise-induced dispersion around the periodic attractor, leading to a quasi-invariant probability density rather than a discrete limit cycle. Thus, while the deterministic dynamics remain simple and strongly confined, the presence of moderate noise induces wide-ranging fluctuations that overshadow the underlying periodicity.

Figure 11b demonstrates a markedly different dynamical regime. With $\lambda_{1}\;=\;-1.0$, $\lambda_{2}\;=\;-0.5$, $\lambda_{3}\;=\;-0.1$, and a stronger forcing amplitude $a_{0}\;=\;3.0$, as well as the initial condition I.C = [2.0, 0.0], the deterministic system develops a highly structured, lobed Poincaré map characteristic of chaotic or near-chaotic dynamics. It is the negative nonlinear coefficients that weaken the restoring force, permitting the excursions of large amplitude and the stretch–fold mechanisms which are the sources of sensitivity to initial conditions. Stochastic excitation forces the complex deterministic geometry to become smoothed to form layered concentric rings of probability: the fine structure of the noise erases the global attractor shape but does not alter the shape of the attractors. This is indicative of a deterministic chaos in a noise-regularized probabilistic structure.

In Figure 11c, the same parameters as in Figure 11b are used, but with the initial conditions shifted to a lower-energy state, i.e, $\lambda_{1}\;=\;-1.0,\;\lambda_{2}\;=\;-0.5,\;\lambda_{3}\;=\;-0.1,\;a_{0}\;=\;3.0,\;$b = 1.2, σ = 0.3, with initial condition I.C = [−1.0, 0.5]. The deterministic Poincare section will then sink into a small periodic loop instead of a chaotic attractor, as was previously seen, which has strong multistability. The oscillator has been kept in a low-energy periodic orbit, and the forcing is not enough to cause the oscillator to move to the chaotic basin. The points are distributed into an arc-shaped band that is tied to the deterministic cycles when stochastic forcing is used. Noise is introduced to the orbit, but unlike in Figure 11b, it does not result in basin bouncing or swings to the chaotic zone. This emphasizes the long-term persistence of the dependence of behavior on the initial conditions, despite having the same parameters.

Figure 11d further highlights multistability by using a different initial condition, again under the same parameter configuration as Figure 11b,c, i.e, $\lambda_{1}\;=\;-1.0,\;\lambda_{2}\;=\;-0.5,\;$$\lambda_{3}\;=\;-0.1,\;a_{0}\;=\;3.0,\;b\;=\;1.2,\;\sigma \;=\;0.3$, with the initial condition I.C = [1.5, 0.0]. In this case, the deterministic dynamics approach a small periodic orbit with an almost circular shape that is more symmetrical than the asymmetric limit cycle in Figure 11c. Its stochastic Poincare map consists of clean concentric rings of probability around the deterministic cycle, which shows that there is a uniform local geometry of the phase-space in which the noise exerts an isotropic action on the system. The stochastic trajectory gives a complete ring, in contrast to the panel of Figure 11c, as a result of the higher rotational symmetry of the attractor. In this case, it is demonstrated that noise can be used to display the invariant statistical structure of a periodic orbit without introducing any distortion in its overall geometry.

### 3.9. Dynamical Characterization via Return Maps and Surrogate Data Validation

The work uses two major methods to describe the dynamics of the system. Return maps transform the continuous-time version into a discrete-time representation, exposing the repetitive nature and aiding in the detection of chaos and periodic orbits. Surrogate data tests are then used to measure whether the observed complexity is due to nonlinear determinism or linear stochastic processes. A combination of these approaches gives a strong, multifaceted diagnosis of the inherent behavior of the system.

**Figure 12:**
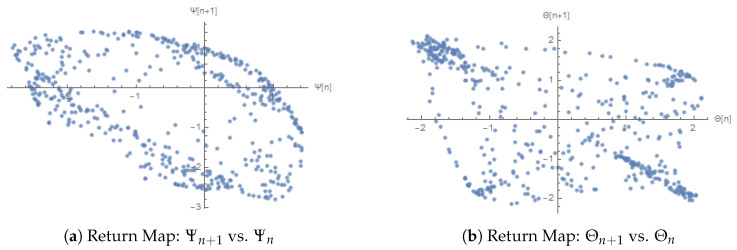
Return maps showing iteration patterns of Ψ and Θ. Sampling is synchronized with the forcing period T=2π/b.

This analysis is supplemented by Figure 12, which shows the 3D trajectories, phase portraits, time series, and return maps of both system variables, namely, Ψ and Θ.

Figure 12a,b showing teh returning maps of Ψ and Θ indicate that there are no clearly defined functional relationships, which once again supports the occurrence of complex and unpredictable behavior. These visualizations of Poincare sections, time series and return maps, together with the evidence of multistability and chaos, are suggestive of sensitivity to initial condition, the coexistence of various types of attractors, and non-periodic orbits in this nonlinear phenomenon.

#### 3.9.1. Delay-Coordinate Reconstruction and Attractor Geometry Analysis

**Figure 13:**
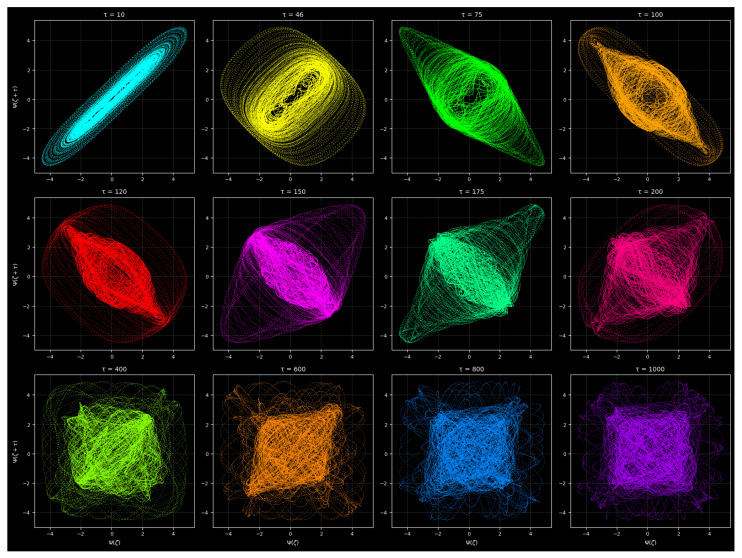
Effect of embedding delay τ on the reconstructed attractor of the forced nonlinear oscillatory System (6). Intermediate delays produce a well-unfolded chaotic attractor, whereas small and large delays yield redundant and de-correlated projections, respectively.

Figure 13 presents a comprehensive delay-coordinate reconstruction of the nonlinear forced cubic oscillator for a wide range of delay values τ. The delay-embedding method is an established tool for analyzing nonlinear time series and for reconstructing the underlying attractor from scalar observations according to Takens’ embedding theorem. In this analysis, the reconstructed attractor is generated using the mapping

(Ψ(ζ),Ψ(ζ+τ)),

where τ is systematically varied to examine how the attractor unfolds at different temporal separations.

The oscillator dynamics are governed by the nonlinear forced System (6), with the parameters chosen as follows:

$$\lambda_{1}\;=\;-0.999,\;\lambda_{2}\;=\;-0.506,\;\lambda_{3}\;=\;-0.18,\;a_{0}\;=\;3.008,\;b\;=\;2.984,$$

and initial conditions

Ψ(0) = 2.0,Θ(0) = 0.

The system is integrated over a long-time interval ζ∈[0, 600] using a high-precision DOP853 integrator with 60,000 output points, ensuring an accurate resolution of its intricate dynamical structure.

Figure 13 displays a grid of twelve subplots corresponding to the following delay values:

τ={10, 46, 75, 100, 120, 150, 175, 200, 400, 600, 800, 1000}.

An obvious development of the attractor morphology appears with an increase in the value of τ, revealing the various dynamical regimes of the oscillator.

##### Small Delays: τ = 10 to τ = 75

At small delay values, there are tightly wound and highly correlated structures in the reconstructed attractor. The plot curve versus τ = 10 indicates an almost one-dimensional, diagonally stretched curve, which implies that there is a high correlation between Ψ(ζ) and Ψ(ζ + τ). After increasing the values of τ to higher values, such as 46 and 75, the attractor starts thickening and exhibiting the multi-loop characteristics, indicating the onset of quasi-periodic or weakly chaotic behavior.

##### Intermediate Delays: τ = 100 to τ = 200

At intermediate delay regimes, the geometry of the attractor grows into larger and more complex forms. The sub-plots of τ = 100–200 have progressively twisted structures with remarkable folding, stretching, and rotational symmetry. These are typical of a chaotic attractor that is sensitive to initial conditions. The attractor begins to fill a two-dimensional space much more uniformly, which means that the values of τ in this range are appropriate for embedding.

##### Large Delays: τ = 400 to τ = 1000

With large delay values, the reconstructed attractor becomes increasingly more de-correlated. The subplots in the case where τ = 400 is used demonstrate the presence of diffuse, square or cloud-like subplots that fill up the phase plane in a largely uniform manner. This spread is due to the fact that, as the delay increases, the behavior of both Ψ(ζ) and Ψ(ζ + τ) increases independently—that is, it progressively increases as the delay increases.

#### 3.9.2. Analysis of Delay Coordinate Reconstruction vs. Surrogate Data

The analysis employs surrogate data testing to learn the difference between true chaotic determinism and the stochastic randomness of a noisy nonlinear oscillator. Our comparison of the original dynamics to phase-randomized surrogates that only maintain linear statistics helps us to understand whether the observed complexity is due to a true nonlinear structure.

**Figure 14:**
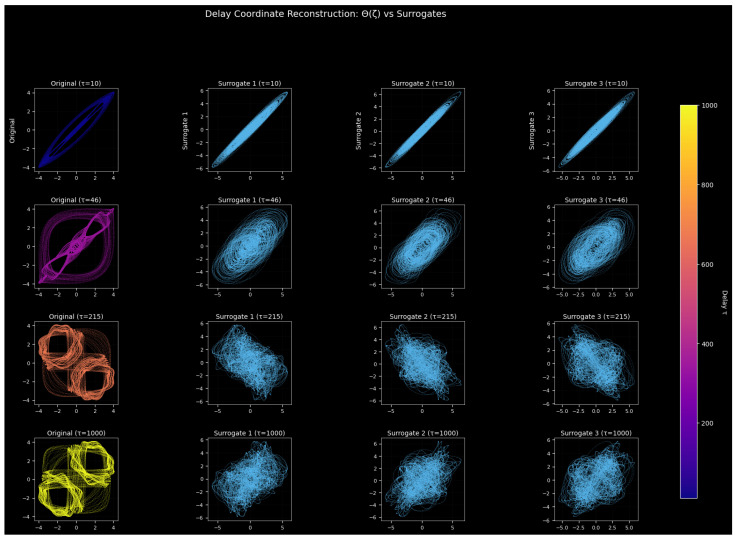
Delay-coordinate reconstructions of the original oscillator signal Θ(ζ) compared with three Fourier-based surrogate datasets across four delay values (τ = 10, 46, 215, 1000). The original data exhibit a coherent geometric structure and multi-lobed attractors, while the surrogates form diffuse clouds, indicating that the observed complexity arises from genuine nonlinear deterministic dynamics rather than purely stochastic linear processes.

Figure 14 shows delay-coordinate reconstructions of the original velocity signal Θ(ζ) and three Fourier-based surrogates for τ = 10, 46, 215, 1000. Fourier-phase randomization leaves variance, autocorrelation, and spectral content constant, and eliminates nonlinear temporal dependencies; hence, the variance in the original versus surrogate embeddings is a sign of nonlinear determinism.

The delay-coordinate reconstructions presented in Figure 14 offer a comparative visualization of the original nonlinear oscillator dynamics against three Fourier transform surrogate datasets across four delay values. The purpose of these reconstructions is to distinguish genuine nonlinear determinism in the measured velocity signal Θ(ζ) from a structure that could arise purely from stochastic processes with similar linear statistical properties. The original data originate from a forced nonlinear oscillatory System (6) governed by a cubic-quadratic restoration force, weak linear damping, external periodic forcing, and additive Gaussian noise. By contrast, the surrogate datasets are constructed to preserve the linear power spectrum and autocorrelation structure of the signal while destroying all underlying nonlinear temporal dependencies. Consequently, differences in the geometric organization between the original and surrogate embeddings provide visual evidence for or against the presence of a deterministic nonlinear structure in the system.

## 4. Conclusions

This work provides a comprehensive analysis of the deterministic and stochastic dynamics of a noise-driven forced nonlinear oscillator in a periodically driven framework. A systematic comparison of three exact analytical traveling-wave solutions under the common parameter set p = 1.0, q = 0.5, κ = 1.0, and γ = 0.4 confirms a consistent nonlinear–dispersive balance across the shared spatiotemporal domain x∈[−3, 3], y∈[−2, 2], and t∈[−1, 1]. The phase-space structure, equilibrium analysis, and bifurcation behavior of System (4) reveal dense, folded, three-dimensional attractors associated with transitions toward quasi-periodic and chaotic regimes, characterized by a sensitive dependence on initial conditions and parameters, amplitude–frequency modulation, and intermittent burst dynamics. The temporal responses of Ψ(ζ) vary significantly with forcing combinations $a_{0}\in \left\{0.8,\;1.2,\;1.6\right\}$ and b∈{1.0, 1.8, 2.5}, where a multilayer attractor indicating periodic or weak chaos contrasts with the strongly chaotic motion confirmed by a positive Lyapunov exponent and state-variable sensitivity. Intermittent bursts in |ΔΘ(ζ)| indicate localized momentum stability governed by nonlinear energy exchange, while the broad multimodal distribution of final Ψ values demonstrates the coexistence of multiple competing attractors and mixed regular–chaotic phase-space structure. Variations in initial conditions further distinguish strange chaotic attractors, invariant-torus quasi-periodicity, intermittent weak chaos, and fully chaotic regimes. Deterministic Poincaré analysis identifies stable periodic confinement for positive nonlinear coefficients, stochastic diffusion into probability clouds under noise, lobed chaotic structures for negative parameter sets, and pronounced multistability, which manifests as small periodic loops with a concentric stochastic probability rings that preserve the invariant geometry, alongside intermittent quasi-periodic–chaotic attractors. The return maps of Ψ and Θ confirm the absence of simple functional dependence and the presence of high-dimensional nonlinear dynamics. Finally, a delay-coordinate reconstruction of the forced cubic oscillator using parameters $\lambda_{1}\;=\;-0.999$, $\lambda_{2}\;=\;-0.506$, $\lambda_{3}\;=\;-0.18$, $a_{0}\;=\;3.008$, and b = 2.984, with initial conditions Ψ(0) = 2.0 and Θ(0) = 0, integrated over ζ∈[0, 600] via a high-precision DOP853 scheme with 60,000 output points, accurately resolves the intricate long-time dynamics. Overall, the oscillator exhibits a rich coexistence of periodic, quasi-periodic, chaotic, and noise-modified behaviors governed by parameter variation, stochastic perturbation, and initial-state sensitivity, offering insight into the stability transitions, energy transfer, and predictability limits in nonlinear driven systems that are relevant to nonlinear physics, engineering oscillators, and stochastic dynamical modeling.

## Data Availability

All relevant data are in the manuscripts.
